# Changes in interoceptive accuracy related to emotional interference in somatic symptom disorder

**DOI:** 10.1186/s40359-024-01778-7

**Published:** 2024-05-17

**Authors:** Deokjong Lee, Se Joo Kim, Jooah Cheon, Young-Chul Jung, Jee In Kang

**Affiliations:** 1 Institute of Behavioral Science in Medicine, Yonsei University College of Medicine, Seoul, 03722 South Korea; 2 Yonsei Empathy Psychiatry Clinic, Seoul, 07008 South Korea; 3 Department of Psychiatry, Yonsei University College of Medicine, Yonsei-ro 50-1, Seodaemun-gu, Seoul, 03722 South Korea; 4 Department of Medicine, Yonsei University Graduate School, Seoul, 03722 South Korea

**Keywords:** Emotional processing, Heartbeat perception task, Heart rate variability, Interoceptive accuracy, Somatic symptom disorder

## Abstract

**Objective:**

The somatic symptom disorder (SSD) is characterized by one or more distressing or disabling somatic symptoms accompanied by an excessive amount of time, energy and emotion related to the symptoms. These manifestations of SSD have been linked to alterations in perception and appraisal of bodily signals. We hypothesized that SSD patients would exhibit changes in interoceptive accuracy (IA), particularly when emotional processing is involved.

**Methods:**

Twenty-three patients with SSD and 20 healthy controls were recruited. IA was assessed using the heartbeat perception task. The task was performed in the absence of stimuli as well as in the presence of emotional interference, i.e., photographs of faces with an emotional expression. IA were examined for correlation with measures related to their somatic symptoms, including resting-state heart rate variability (HRV).

**Results:**

There was no significant difference in the absolute values of IA between patients with SSD and healthy controls, regardless of the condition. However, the degree of difference in IA without emotional interference and with neutral facial interference was greater in patients with SSD than in healthy controls (*p* = 0.039). The IA of patients with SSD also showed a significant correlation with low-frequency HRV (*p* = 0.004) and high-frequency HRV (*p* = 0.007).

**Conclusion:**

SSD patients showed more significant changes in IA when neutral facial interference was given. These results suggest that bodily awareness is more affected by emotionally ambiguous stimuli in SSD patients than in healthy controls.

## Introduction

The DSM-5’s proposed diagnostic criteria for somatic symptom disorder (SSD) include distressing somatic symptoms and related excessive thoughts, feelings, or behaviors [1]. Although the underlying mechanisms of somatic symptoms in SSD have not been fully clarified, altered perception of bodily signals has been suggested to play important role in the disorder’s pathophysiology [2]. Some previous studies of patients with somatoform disorder have reported excessive recognition of bodily signals related to somatic symptoms [3], whereas other such studies have indicated diminished perception of internal bodily sensations [4]. Although these results seem to be conflicting, the selective attentional shift from normative bodily signals to somatic symptoms has been presented as a possible explanation [5]. This abnormal attentional focus on somatic symptoms in SSD has been proposed as an automatic process performed according to previously formed memory structures (schemata) for somatization.

Interoceptive accuracy (IA), or the difference between subjective estimation and objective measurement of an internal bodily state, is an important indicator of one’s ability to appropriately perceive one’s bodily signals [6]. Behavioral tasks that measure how accurately a person can estimate their heart rate (the mental tracking paradigm) [7] and distinguish their heartbeat from external stimuli (the heartbeat discrimination paradigm) [8] have been used to assess IA. Previous studies using these tasks on patients with somatoform disorder showed that IA in these patients was similar to that of normal controls [9, 10]. However, in situations where other psychological factors were involved, the IA of individuals with somatization disorders tended to decline [4, 11]. We speculate that IA in SSD patients does not simply increase or decrease but is distorted by other psychological factors that may affect the patients’ schemata for somatization.

Somatization has long been regarded as a defensive mechanism in which intolerable emotional conflicts are converted into somatic complaints. Consistent with this concept, several previous studies have suggested that disturbance of emotional processing is one of the most crucial psychopathological factors in SSD [12]. Other studies have demonstrated that patients with somatoform disorders exhibited difficulties with emotional awareness [13] and recognition of facial emotions [14]. A recent study of SSD patients found that while SSD patients have difficulty recognizing their own emotions, they are more sensitive to the negative emotions of others [15]. The high comorbidity of somatoform disorder and depression also implies a strong association between somatization and emotional processing [16]. Taken together, we speculate that patients with SSD have difficulty processing emotional information and that these difficulties may substantially affect their clinical features. This view is supported by previous findings, which have shown that disturbed autonomic nervous system activity in SSD was more remarkable when emotional processing was engaged [11]. We also speculate that IA alteration is one of the core features related to the pathophysiology of SSD, which would be affected by whether or not emotional processing is involved. Previous studies indicating that IA is affected by emotional state and is related to the emotional regulation, such as reappraisal, support our speculation [17, 18].

This study aimed to examine whether patients with SSD struggle with the perception of bodily signals and, specifically, how this perception is impacted by emotional processing. We hypothesized that patients with SSD would show significant differences in IA when emotional processing was involved. To test our hypothesis, we compared the IA of SSD patients with that of healthy controls using the mental tracking paradigm. In particular, we selected facial interference as an emotional processing stimulus and observed the degree of change in IA after presenting the stimulus. Facial stimuli have been widely used in psychological tasks related to emotion recognition and emotional responding [19, 20]. We also speculated that IA, as measured through the mental tracking paradigm, could be significantly correlated with the clinical characteristics of SSD.

## Methods

### Participants

We recruited patients who were clinically diagnosed with SSD according to the DSM-V diagnostic criteria in a psychiatric outpatient clinic at Severance University Hospital in Seoul. Healthy controls were recruited through online advertising, flyers, and word of mouth. The exclusion criteria applied to all participants in this study were as follows: major psychiatric disorders other than SSD, low cognitive function that would cause difficulty in completing a self-reported questionnaire, medical problems that directly lead to physical symptoms, and current medical treatment with medication. A certified psychiatrist conducted interviews to confirm that the patient group had no major psychiatric disorders besides somatization disorder and the control group had no major psychiatric disorders.

Finally, 17 female and 6 male SSD patients (age = 33.9 ± 10.1 years) and 14 female and 6 male healthy controls (age = 30.6 ± 8.1 years) were included in the study. The group of healthy controls also participated in our previous study using different behavioral tasks (dot-probe task) [21]. The protocols used in this study were approved by the Institutional Review Board at Severance Hospital, Yonsei University, South Korea, and all subjects were provided detailed explanations of the study and signed a consent form before participating.

### Measurement of clinical characteristics

All subjects were given several self-report rating scales to assess their clinical characteristics. The severity of their somatic symptoms was assessed through the somatization subscale of the Symptom Checklist-90-Revised (SCL-90-R) [22]. The amplification of somatosensory signal recognition was evaluated using the Somatosensory Amplification Scale (SSAS) [23]. The degree of difficulty in recognizing emotions was assessed using the Toronto Alexithymia Scale (TAS) [24]. The Center for Epidemiologic Studies Depression (CES_D) scale [25] was used to assess depressive symptoms, and the State-Trait Anxiety Inventory state instrument (STAIX_S) [26] was used to assess anxiety symptoms.

Resting-state heart rate variability (HRV) was also calculated for all subjects using a 5-minute electrocardiogram (ECG) measurement. The detailed methods of HRV measurement were described in our previous study of SSD patients [21]. We established frequency domains for the HRV parameters, i.e., high-frequency (HF)-HRV and low-frequency (LF)-HRV and transformed the parameters with the natural logarithm.

### Measurement of interoceptive accuracy

**Fig. 1:**
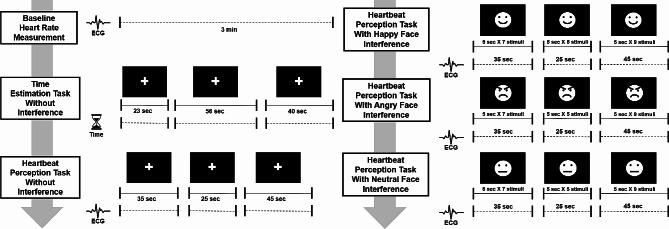
Experimental process of the mental tracking paradigm in the current study. In the diagram, face photo stimuli were replaced with emotional face icons. The photo stimuli actually used in this study were selected from the Korean Facial Expressions of Emotion (KOFEE) developed by Park JY et al., and an example photos can be found in the following paper: Lee SB, Koo SJ, Song YY, Lee MK, Jeong Y-J, Kwon C, et al. Theory of mind as a mediator of reasoning and facial emotion recognition: findings from 200 healthy people. Psychiatry Investigation. 2014;11(2):105

We used the mental tracking paradigm adjusted by Ehlers and Breuer [27] to measure the IA of this study’s subjects. This paradigm assesses whether the subjects can count their heartbeats (the heartbeat perception task) and estimate the passage of time (the time estimation task). The experimental process of the mental tracking paradigm implemented in this study is depicted in Fig. 1. The experiment in this study consisted of baseline heart rate measurement, a time estimation task, and four heart rate estimation tasks. The heartbeat perception tasks were conducted in the following order: no interference, happy facial interference, angry facial interference and neutral facial interference. The order of each condition in the heartbeat perception task was the same for all subjects.

Before the tasks were performed, each subject’s baseline heart rate was measured during a 3-minute rest period. In the heartbeat perception task, subjects were instructed to quietly focus on their bodily sensations and count their heartbeats without a direct measurement (e.g., taking their pulse) between two beeps. An ECG was conducted to calculate the actual heart rate after the task. In the time estimation task, the subjects were instructed to estimate how many seconds passed between the two beeps, and we prevented them from performing actions that could directly measure time. The time estimation task was performed to confirm that the subjects’ ability to recognize the flow of time was not a confounding variable in counting their heartbeats. The heartbeat perception task consisted of three trials that were run for 35, 25, and 45 s. The time estimation task consisted of three trials that were run for 23, 56, and 40 s. Heartbeat perception and time estimation error scores were calculated by subtracting the estimated value from the actual value and dividing the result by the actual value. The mean error scores for heartbeat perception and time estimation were calculated by averaging the values obtained from the three trials. The accuracy scores for heartbeat perception and time estimation were calculated by subtracting the mean error scores from 1 and converting them to percentages.

### Interoceptive accuracy under emotional interference

To determine the effect of the emotional interference, we measured the subjects’ IA using the heartbeat perception task during emotional interference. Emotional facial photographs have been widely used in previous research for this purpose [28, 29]. Furthermore, the brain’s response to emotional faces is influenced more by the emotional information contained in the face rather than the attentional process [30]. We used facial photo stimuli from the Korean Facial Expressions of Emotion (KOFEE) photographs as emotional interference in the current study [31]. Examples of photo stimuli used in this study are presented in previous research papers using the KOFEE [32, 33]. Five male and five female faces, each with happy, angry, and neutral expressions, were chosen from the set. We used photographs that did not show hair features or clothing to minimize the impact of physical appearance on the results. Emotional interference was presented as one of the facial photographs in the center of an otherwise black screen. During the heartbeat perception task with emotional interference, each subject was asked to focus on their body’s senses as they looked at the photographs.

The heartbeat perception task consisted of three trials (35, 25, and 45 s) for each condition (happy, angry, and neutral expressions). Throughout the three trials of each condition, 21 facial photographs of 10 people were used as interference stimuli. Each person’s photograph was used twice, but the first photograph presented was used once more at the end. Each photographic stimulus was presented for 5 s, and the next photographic stimulus was presented without an intermission. The order in which photographic stimuli were presented was the same for each condition (happy, angry, and neutral expressions) and for all subjects. At the end of each trial, subjects were asked to guess how many times their heart had beat, and their IA was calculated. The IA values of the three trials for each condition (happy, angry, and neutral faces) were averaged.

### Statistical analysis

Statistical analyses were performed using the Statistical Package for the Social Sciences (SPSS), version 25.0 K (SPSS Inc., Chicago, IL, USA). *P*-values less than 0.05 were considered statistically significant. We applied independent t-tests and χ^2^ tests to compare demographic data and clinical characteristics between patients with SSD and healthy controls. Differences in time estimates and IA between these groups were verified by independent t-tests.

To identify group differences in the influence of interfering stimuli on IA, the two-way repeated measures analysis of variance (ANOVA) was performed in order to determine whether there is a significant interaction between group (SSD patients, healthy controls) and condition (no interference, with happy facial interference, with angry facial interference, with neutral facial interference) on IA score. The Bonferroni correction was applied to post-hoc analysis. A priori power analysis was performed using G*Power 3.1 software [34]. It revealed a sample size of 36 participants (a minimum of 18 subjects in each group) to potentially detect the global effect of the repeated measure ANOVA with a medium effect size of 0.25 and a power of 0.95.

To individually analyze the impact of each emotion type on IA in line with the hypotheses of this study, the difference between the IA value of each interference condition and the IA value without interference was calculated and compared between groups. Additionally, analysis of covariance (ANCOVA) was also performed by entering CES_D and STAIX_S as covariates to control for the influence of affective symptoms.

To explore the relationship between IA and other clinical variables, we also analyzed the correlation between IA (without interference) and clinical variables (SCL-90-R, SSAS, TAS, HF-HRV, LF-HRV) within each group (SSD patients, healthy controls). The partial correlation analysis controlled for CES_D and STAIX_S.

## Results

### Demographic and clinical characteristics of participants

Age and gender were not significantly different between the patients with SSD and controls. (Table 1). We found no significant differences in mean baseline heart rate between patients with SSD and healthy controls during the time estimation task. In the heartbeat perception task, there was no significant difference in heart rate between SSD patients and controls both in the absence of interfering stimuli and in the presence of any type of interfering stimuli. Patients with SSD scored significantly higher on somatic symptom severity and somatosensory amplification (somatization subscale of the SCL-90-R: *t* = 4.531; *p* < 0.001; SSAS: *t* = 3.099, *p* = 0.004). Alexithymia was significantly higher in patients with SSD than in the controls (TAS: *t* = 3.157, *p* = 0.003), as were depression and anxiety symptoms (CES_D: *t* = 3.527, *p* = 0.001; STAIX_S: *t* = 5.168, *p* < 0.001). Patients with SSD had lower HRV parameters than healthy controls (LF-HRV: *t*=-2.502, *p* = 0.016; HF-HRV: *t*=-2.242, *p* = 0.030).

**Table 1:** Demographics and physiological and clinical variables of participants

	SSD patients (*n* = 23)	Controls (*n* = 20)	Test	*p*-value	Effect Size
**Demographic Variables**					
Sex (male), numbers (%)	6 (26.1)	6 (30.0)	χ^2^ = 0.081	0.775	0.044
Age, years	33.9 ± 10.1	30.6 ± 8.1	1.178	0.246	0.360
**Physiological Variables**					
Baseline Heart Rate, beats per minute	76.6 ± 11.9	73.7 ± 11.3	0.811	0.422	0.248
Heart Rate During Heartbeat Perception Task					
Without Interference	77.6 ± 20.9	70.6 ± 7.7	1.503	0.144	0.435
With Happy Face Interference	79.0 ± 23.3	69.7 ± 7.5	1.545	0.132	0.365
With Angry Face Interference	77.6 ± 22.3	71.1 ± 7.9	1.305	0.202	0.378
With Neutral Face Interference	79.6 ± 23.2	72.9 ± 7.5	1.302	0.204	0.376
**Clinical Variables**					
SCL	27.6 ± 10.4	17.1 ± 3.7	4.531	< 0.001	1.311
SSAS	22.8 ± 6.2	17.7 ± 4.1	3.099	0.004	0.947
TAS	54.4 ± 9.9	45.2 ± 9.1	3.157	0.003	0.965
CES_D	24.6 ± 6.3	18.8 ± 4.6	3.527	0.001	1.055
STAIX_S	55.1 ± 10.8	38.7 ± 10.0	5.168	< 0.001	1.580
**Heart Rate Variability**					
Low Frequency	4.9 ± 1.0	5.6 ± 1.0	−2.502	0.016	-0.765
High Frequency	5.0 ± 1.1	5.7 ± 1.0	−2.242	0.030	-0.685

*CES_D* Center for Epidemiologic Studies Depression scale, *SCL-90-R* Symptom Checklist-90-Revised, *SSAS* Somatosensory Amplification Scale, *SSD* somatic symptom disorder, *STAIX_S* State-Trait Anxiety Inventory state instrument, *TAS* Toronto Alexithymia Scale

Data are expressed as mean ± standard deviation unless otherwise indicated

All *p*-values were calculated with the independent t-test or χ^2^ test

As for effect size, the w value was applied only to gender, and Cohen’s d was applied to other variables

### Interoceptive accuracy measurements

We found no significant differences in the estimation of time or perception of heartbeats between patients with SSD and healthy controls in the absence of interference stimuli (Table 2). The heartbeat perception task using any type of facial interference also showed no significant difference in IA between patients with SSD and healthy controls. According to post-hoc power analysis, power ranged from 0.3 to 0.5 for group comparisons in the heartbeat perception task with facial interference (happy face: 0.360; angry face: 0.300; neutral face: 0.404).

**Table 2:** Interoceptive accuracy in the heartbeat perception tasks

	SSD patients (*n* = 23)	Controls (*n* = 20)	Test	*p*-value	Effect Size
Time Estimation Task, %	66.5 ± 17.5	71.1 ± 19.6	−0.812	0.421	-0.248
**Heartbeat Perception Task**					
Without Interference (R), %	57.1 ± 20.6	57.2 ± 24.7	−0.017	0.987	-0.005
With Happy Facial Interference (H), %	46.7 ± 17.9	56.9 ± 23.1	−1.623	0.112	-0.496
With Angry Facial Interference (A), %	53.1 ± 17.8	62.6 ± 24.5	−1.427	0.163	-0.446
With Neutral Facial Interference (N), %	46.7 ± 18.5	57.5 ± 22.2	−1.733	0.091	-0.530
Difference Between H and R, %	−10.4 ± 19.7	−0.3 ± 14.1	−1.897	0.065	-0.580
Difference Between A and R, %	−4.0 ± 16.8	5.3 ± 16.0	−1.854	0.071	-0.567
Difference Between N and R, %	−10.4 ± 19.9	0.3 ± 10.8	−2.131	0.039	-0.652

*SSD* somatic symptom disorder

Data are expressed as mean ± standard deviation

All *p*-values were calculated with the independent t-test

Cohen’s d was used to indicate effect size

**Fig. 2:**
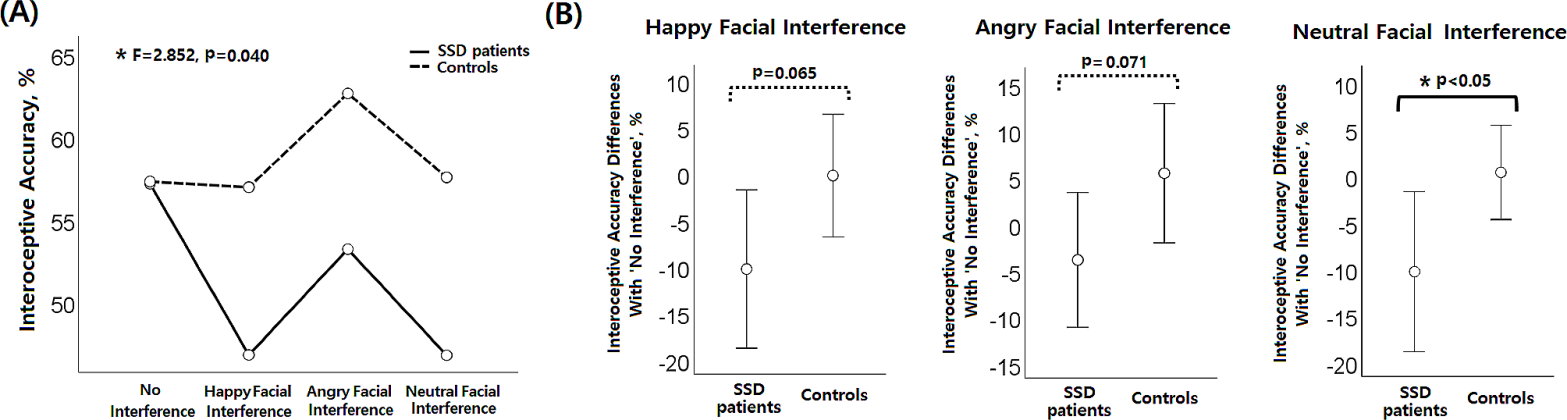
Comparison of the heartbeat perception task results between somatic symptom disorder (SSD) patients and healthy controls. (**A**) Repeated measures analysis of variance (ANOVA) results. (**B**) Comparison of SSD patients and controls on the difference in interoceptive accuracy (IA) with and without emotional interference. The IA value in the ‘with neutral face interference’ condition minus the IA value in the ‘no interference’ condition revealed a significant statistical difference between SSD patients and controls

Figure 2 depicts the results of a comparison of the heartbeat perception task between SSD patients and healthy controls. The interaction effect between group (SSD patients, healthy controls) and condition (no interference, with happy facial interference, with angry facial interference, with neutral facial interference) on IA was significant (*F* = 2.852, *p* = 0.040). The main effect of group was not significant (*F* = 1.651, *p* = 0.206), but the main effect of condition was significant (*F* = 4.674, *p* = 0.004). The post-hoc paired comparison showed that the differences between conditions ‘with angry facial interference’ and ‘with happy facial interference’ (*t* = 3.547, *p* < 0.001), and between conditions ‘with angry facial interference’ and ‘with neutral facial interference’ were significant (*t* = 3.774, *p* < 0.001). Even when CES_D and STAIX_S were entered as covariates, the interaction effect between group and condition was significant (*F* = 6.294, *p* = 0.016), but the main effect of each of group and condition was not significant (group: *F* = 0.347, *p* = 0.559; condition: *F* = 2.054, *p* = 0.160).

The degree of difference in IA without emotional interference and with neutral facial interference was greater in patients with SSD than in healthy controls (*t*=-2.131, *p* = 0.039). The degree of difference in IA in the absence of facial interference and in the presence of happy or angry facial interference showed a trend-level difference between patients with SSD and healthy controls (happy face: *t*=-1.897, *p* = 0.065; angry face: *t*=-1.854, *p* = 0.071). Post hoc power analysis showed that the power for group comparisons of the degree of IA difference between those with and without facial interference (happy faces: 0.542, angry faces: 0.459, neutral faces: 0.606) ranged from 0.4 to 0.7. Additionally, in ANCOVA with CES_D and STAIX_S entered as covariates, group differences were significant for all types of facial interference (happy face: *F* = 4.402, *p* = 0.042; angry face: *F* = 5.344, *p* = 0.026; neutral face: *F* = 6.048, *p* = 0.018).

### Correlation analysis

LF-HRV and HF-HRV showed a significant positive correlation in both groups (patients with SSD: *r* = 0.766, *p* < 0.001; healthy controls: *r* = 0.656, *p* < 0.001; Table 3). In patients with SSD, lower IA was significantly correlated with lower HRV parameters (LF-HRV: *r* = 0.614, *p* = 0.003; HF-HRV: *r* = 0.604, *p* = 0.004). There was no significant correlation between IA and HRV parameters in healthy controls.

**Table 3:** Partial correlation matrix for interoceptive accuracy and clinical variables (the Center for Epidemiologic Studies Depression scale and State-Trait Anxiety Inventory state instrument were controlled)

Variables	IA	SCL	SSAS	TAS	LF	HF
**SSD patients (n = 23)**						
*IA*	1					
*SCL*	0.044	1				
*SSAS*	0.086	0.033	1			
*TAS*	0.288	0.067	0.125	1		
*LF*	0.614**	0.153	−0.244	0.062	1	
*HF*	0.604**	0.259	−0.021	0.072	0.766***	1
**Controls (n = 20**						
*IA*	1					
*SCL*	0.238	1				
*SSAS*	−0.195	−0.434	1			
*TAS*	−0.259	−0.236	0.170	1		
*LF*	0.238	−0.035	−0.435	0.023	1	
*HF*	0.379	0.035	−0.191	0.181	0.656**	1

*HF* high frequency, *IA* interoceptive accuracy, *LF* low frequency, *SCL-90-R* Symptom Checklist-90-Revised, *SSAS* Somatosensory Amplification Scale, *SSD* somatic symptom disorder, *TAS* Toronto Alexithymia Scale

## Discussion

This study compared the body perception of SSD patients with that of healthy controls by using the mental tracking paradigm to measure IA. There was no statistically significant difference in IA between the SSD patients and healthy controls regardless of the absence or presence of emotional interference. These results are consistent with previous studies that showed similar IA between SSD patients and healthy controls [10]. However, repeated measures ANOVA showed that the impact of facial interference on IA in SSD patients differed from that in healthy controls depending on the type of emotion involved. In particular, the difference in IA in the absence of interference and in the presence of neutral facial interference was more pronounced in SSD patients than in healthy controls. Previous studies have reported that neutral face photographs may be perceived as containing emotions in the experimental environment [35]. Therefore, our current findings are partially consistent with our hypothesis that patients with SSD have difficulty recognizing bodily signals when emotional processing is involved.

In this study, only the effect of neutral facial interference on IA was significant; the effects of happy or angry facial interference on IA were non-significant. Previous research has found that neutral faces were interpreted as ambiguous stimuli containing emotion rather than as stimuli without emotion [35]. When people look at another person’s face, they tend to automatically extract the emotional meaning behind that facial expression, even if that facial expression does not display any emotion [36]. Patients with somatoform disorder have been found unable to accurately distinguish emotions from facial expressions [14]. As in the sample of this study, patients with SSD have a high tendency for alexithymia [37], which is associated with their impaired ability to perceive the emotional facial expressions [38]. Therefore, we speculate that SSD patients may have experienced a greater burden of emotional processing when presented with ambiguous (neutral facial interference) rather than clear emotional stimuli (happy or angry facial interference). Interoceptive representations, formed through the integration of internal bodily signals, are the basis for emotional awareness and other emotional processes [39, 40]. Recent predictive coding models of interoception suggested that prediction error signal occurs through comparison of descending interoceptive predictions and ascending interoceptive neural signals [41, 42]. This interoceptive prediction error signal has been related to emotions and selfhood [43, 44]. We speculate that the current finding of reduced IA for neutral facial interference may be related to an altered framework of interoceptive predictive coding in SSD. We also speculate that the difficulties with interoceptive prediction may be heightened in SSD patients because neutral faces are perceived as emotionally ambiguous. These speculations should be verified through future studies, including comparisons between SSD patients and controls on how they perceived emotions for neutral faces.

Under the interpretation of neutral facial interference as stimuli related to emotional processes, our current results suggest that patients with SSD have difficulty handling emotional stimuli. This suggestion is consistent with the psychoanalytic explanations of somatization that individuals with somatization are unable to adequately deal with emotional information [12, 45]. Accumulating research on somatoform disorders has consistently suggested impaired emotional regulation in subjects with somatoform disorders [46, 47]. This emotional dysregulation may be related to maladaptive defense mechanisms, and previous studies have suggested that defense mechanisms such as suppression and displacement of negative emotions are associated with the formation of somatic symptoms [48–50]. When future studies explore the relationships between interoception, somatization, and emotional processing, it would also be helpful to explore subjects’ psychological defense mechanisms to better understand the development of somatic symptoms.

Additionally, the results of this study can be interpreted from the perspective of trustworthiness. We automatically form trustworthiness of other individuals through bottom-up emotion-attention interactions from facial features [51]. Previous studies reported that identifying trustworthiness from neutral facial stimuli is related to the function of the autonomic nervous system [52, 53]. SSD patients have been found to show alterations of the autonomic nervous system and low levels of trustworthiness [15]. Taken together, the distinct changes in IA for neutral facial interference in this study may have been influenced by the differences in the propensity of trustworthiness readings from facial stimuli of SSD. This interpretation could be tested through further studies with psychological experimental designs involving trustworthiness.

In this study, there were no significant differences in the degree of influence on IA for happy or angry facial interference between SSD patients and controls. However, there were group differences at the trend level, which is partially consistent with the hypothesis that engagement in emotional processing may influence IA in patients with SSD. Additionally, when depressive and anxiety symptoms were controlled as covariates, there were significant group differences in the degree of IA change between no interfering stimuli and interfering happy or angry faces. These results suggest that there may be different interoceptive responses to specific emotional stimuli depending on the presence or absence of coexisting affective symptoms. Therefore, in future studies, it will be necessary to target a larger number of SSD patients and analyze them by dividing them into subgroups according to the presence or absence of accompanying affective symptoms.

SSD patients in this study showed lower HRV than the controls, suggesting that they experienced autonomic nervous system alterations. The HRV of SSD patients also showed a significant correlation with IA. These results are consistent with recent studies that have reported the relationship between HRV and IA [54]. The connection between mental processes and internal bodily state has been shown to utilize the autonomic nervous system functions [55]. Previous studies have suggested that the autonomic nervous system plays an important role in regulating interception through descending neural pathways [56]. Therefore, the current HRV findings suggest that alterations in the autonomic nervous system in patients with SSD contributed to distortions in the perception of bodily signals. Additionally, according to the neurovisceral integration model, HRV is known to reflect not only autonomic nervous system activity but also connectivity with the prefrontal cortex including the ventromedial prefrontal cortex [57]. The prefrontal cortical network, centered on the ventromedial prefrontal cortex, plays a major role in the regulation of interoceptive signals for dynamic interactions with the environment [58, 59]. Although this study did not assess features of prefrontal cortex, our findings reporting HRV changes in SSD are consistent with previous studies reporting ventromedial prefrontal alterations in somatoform disorders, including pain disorders [60–62]. In addition, previous studies have reported on the close associations between IA, HRV, empathy and emotion recognition [63–66]. Therefore, the correlation between IA and HRV specifically found in SSD patients supports that the pathophysiology of SSD is associated with IA and emotional processing. Future research, including investigation of emotional processing such as empathy and emotion recognition, will help to further expand the results of this study.

There are several limitations to consider when interpreting our findings. First, this study had few participants, making it difficult to derive significant, generalizable results. Post-hoc power analysis of the independent t-test conducted in this study showed that the power value was less than 0.8. This suggests that negative findings may have been made because the sample size of the subjects was insufficient. However, the SSD patients were relatively homogenous with no other major psychiatric illnesses, and the patient and control groups were demographically well matched. Second, this study measured IA using only one paradigm. Although the tasks used in this study proved useful in several previous studies, many complex factors could affect one’s ability to recognize one’s bodily sensations. Using a variety of tools, including the heartbeat discrimination paradigm, will help reinforce our results. Third, this study evaluated subjects’ IA through heartbeat perception task, which assessed how accurately they calculated their heart rate. Therefore, whether subjects knew their usual pulse rate may have influenced the results of the heartbeat perception task. Since this study did not include an investigation into this, this may be a limitation of this study. Fourth, this study did not include threat and fear stimuli as emotional interference. Previous studies have suggested that baroreceptor signaling is closely related to threat processing [67], so cardiac interoceptive effects are particularly shown for threat and fear stimuli [68]. Fifth, because the same paradigm was repeatedly performed, the results may have been impacted by the learning effect rather than the task condition alone. This study showed that IA in the condition with neutral facial interference was reduced compared to the condition without interference in patients with SSD, even though the heartbeat perception task using neutral facial interference was the last task. This suggests that despite the limitations of this study design, the main findings of this study may still be valid. However, another possibility is that when performing the same behavioral task repeatedly, the level of learning effect may be different between the patient and control groups. In future studies, randomizing the different conditions of the heartbeat perception task would help rule out the influence of learning effects.

In conclusion, patients with SSD showed more pronounced IA changes compared with healthy controls when neutral facial interference was given. The results of this study suggest that the perception of bodily signals in patients with SSD is not simply increased or decreased as a whole but may be influenced by various psychological factors, including emotionally ambiguous interference. Our current findings imply that disturbed emotional processing may contribute to the development and maintenance of somatic symptoms in SSD.

## Data Availability

The datasets generated during and/or analyzed during the current study are available from the corresponding author on reasonable request.
